# Evaluation of the Training Program of the Project P.A.T.H.S.: Findings Based on the Perspective of the Participants from Different Cohorts

**DOI:** 10.1100/2012/687198

**Published:** 2012-04-01

**Authors:** Daniel T. L. Shek, Yammy L. Y. Chak

**Affiliations:** ^1^Department of Applied Social Sciences, The Hong Kong Polytechnic University, Hong Kong; ^2^Public Policy Research Institute, The Hong Kong Polytechnic University, Hong Kong; ^3^Department of Social Work, East China Normal University, Shanghai 200000, China; ^4^Kiang Wu Nursing College of Macau, Macau; ^5^Division of Adolescent Medicine, Department of Pediatrics, Kentucky Children's Hospital, University of Kentucky College of Medicine, Lexington, KY 40506, USA

## Abstract

Subjective outcome evaluation findings based on the perspective of the participants participating in a 3-day training program of the Project P.A.T.H.S. are reported in this paper. The findings were based on the data collected from the training workshops conducted between 2005 and 2009 (*N* = 4.167). Results showed that the respondents had good and positive perceptions of the training program and found it very valuable, particularly with respect to training instructors and familiarization with the project. Besides, the training participants were able to acquire attitude, knowledge and skills that are conducive to the successful implementation of the program. Based on the subjective outcome evaluation findings, it is concluded that the training program was effective in helping the participants to acquire the necessary knowledge, attitudes and skills in implementing the program.

## 1. Introduction

To promote positive and holistic development of adolescents in Hong Kong, The Hong Kong Jockey Club Charities Trust provided an earmarked grant of HK$ 750 million (HK$ 400 M for the first cycle and HK$ 350 M for the second cycle) to initiate and support the development, implementation, and evaluation of a positive youth development program entitled “P.A.T.H.S. to Adulthood: A Jockey Club Youth Enhancement Scheme” for junior secondary school students. The Project P.A.T.H.S. is a ground-breaking, indigenously developed, multiyear positive youth development program in Chinese context. There are two tiers of the programs, with the aims to promote holistic development of adolescents by providing opportunities and recognition for them to develop competence and skills which are conducive to positive youth development, promoting bonding with others, and holding healthy beliefs and clear values. The Tier 1 Program is a multi-year universal program designed for all Secondary 1 to Secondary 3 students based on 15 positive youth development constructs identified from the existing successful positive youth development programs [1, 2], while the Tier 2 Program is designed for students with greater psychosocial needs. With the support of the education and social welfare sectors, almost half of all the secondary schools in Hong Kong have joined the Project P.A.T.H.S. since its launch in 2005. From 2005 to 2009, there were altogether 223,101 students participating in the Tier 1 Program. Curricula for Secondary 1, Secondary 2, and Secondary 3 students were developed and tailored for Chinese adolescents by the research team comprising academics from five universities in Hong Kong. 

### 1.1. Training Program of the Project P.A.T.H.S

In different fields, substantial time, money, man power, and energy are usually invested in in-service training programs. It is generally believed that investments in in-service training are associated with individual and organization benefits. In the same vein, in-service teacher training is commonly seen as one of the means to help teachers continue to acquire and enhance their professional knowledge and skills [3–6]. In addition, professional development can help enhance teacher performance and improve the learning and achievement of the students [3, 7, 8]. Guskey [9] pointed out that “high-quality professional development is a central component in nearly every modern proposal for improving education” (page 381). 

In Hong Kong, there are various in-service training programs offered for teachers and social workers, which are mainly organized and conducted by the Education Bureau, Social Welfare Department, and other nongovernmental organizations. Like many practitioners in other fields, teachers and social workers may be unenthusiastic to adopt new programs or practices (as they may need to use a very different instructional approach during the implementation of new program) unless they feel secure and confident that they can make them work. Frontline experiences show that teachers' confrontation might hamper the implementation of a new program. It is believed that the provision of pre-implementation training can increase the confidence of the program implementers. Veenman et al. [6] suggested that in-service training can serve three main purposes: “(1) to stimulate the professional competence and development of teachers; (2) to improve school practice; and (3) to implement political agreed-upon innovations in schools” (page 303). To ensure a smooth and effective implementation of the Tier 1 Program of the Project P.A.T.H.S., pre-implementation training for the program implementers is of great importance and the investment of in-service training is considered to be justified. 

Systematic and training programs were designed and offered to all participating schools in the P.A.T.H.S. Project. The program implementers (teachers and/or social workers) involved in the Project were invited to participate in a 3-day training workshop, consisting of 20 hours of training for each grade (Secondary 1 to Secondary 3). Each training workshop provided 12 sessions of training, which were held in 3 days within the same week. The 3-level progressive training program has six general objectives which to help the potential program implementers: (a) to understand the nature of adolescent development and the related issues and to cultivate a positive attitude to adolescent development; (b) to understand the nature of positive youth development, including its basic concepts, related programs, and research; (c) to familiarize themselves with the nature of Project P.A.T.H.S., including its basic philosophy, design, implementation, and evaluation; (d) to understand the content of the Tier 1 Program, including the core program and elective program; (e) to acquire the attitude, knowledge and skills that are conducive to the successful implementation of the Tier 1 Program; (f) to establish a self-help support network among the program participants [10]. The six objectives were addressed throughout the training workshops. Through interactive training, potential program implementers can recognize how to deliver the programs effectively. It was also expected that participants' motivation and sense of ownership of the program would be developed and hence enhancing successful program implementation.

According to Shek and Chak [10], the training programs of the Project P.A.T.H.S. have several unique features. First, progressive training programs are tailored for the program implementers of Secondary 1 to Secondary 3 levels, stage by stage from introductory level (Secondary 1) to advanced level (Secondary 3). Second, a 3-day program at each grade is designed. The first day (Day 1) gives the background of the project, which includes perspectives on adolescent development, positive youth development, project design, program implementation issues, and evaluation mechanisms. Day 2 and Day 3 of the training focus on discussing the related teaching methodologies and units in the Tier 1 Program with reference to different positive youth development constructs. In addition, ways to promote worker efficacy and reflection are included. Third, the training was experiential in nature with teachers actively engaged in all the lessons of the curriculum as participants, thereby demonstrating participatory and facilitative learning. Fourth, open discussion and interaction is emphasized in the training programs. Fifth, conscious effort is made to promote the passion and involvement of the potential program implementers. Finally, reflective learning is strongly emphasized in the training programs.

## 2. Evaluation of the Training Program

To appraise and determine the effectiveness of the training programs, program evaluation is indispensable. Scriven [11] defined evaluation as “the process of determining the merit, worth, and value of things and evaluations are the products of that process” (page 1). Guskey [12] emphasized the necessity of the existence of evaluation in professional development, especially in education sector. Although different in-service training programs are regularly provided for teachers and social workers, most of the developers of in-service training did not pay much attention to evaluation and thus very few programs have been empirically evaluated or documented [13]. Without such evaluation, it is not easy to determine if the training is beneficial to the training participants and their students or clients. At the same time, as published work on positive youth development training programs in Chinese communities is virtually, it is very important to document the details of the training programs. Against this background, this paper documents the evaluation findings related to 121 training workshops carried out during the Experiential Implementation Phase and Full Implementation Phase of the Project P.A.T.H.S. for the Secondary 1, Secondary 2, and Secondary 3 levels from school year 2005 to 2009.

Definitely, the training participants must be engaged in the evaluation process and their voices should be heard. As stated by the Joint Committee on Standards for Educational Evaluation [14], stakeholders should be identified (Standard U1) and the evaluation should take into account the different views of the stakeholders (Standard F2). According to utilization-focused evaluation proposed by Patton [15], significant stakeholders should be included in the evaluation process. When evaluating training effectiveness, numerous methods may be utilized. Obviously, one of the most common and direct ways to evaluate and determine the effectiveness of a training program is to understand the subjective views and reaction of the program participants (i.e., subjective outcome evaluation or client satisfaction approach). Regarding the outcome evaluation of training program, the classic four-level training evaluation model proposed by Kirkpatrick [16, 17] is dominant and well denoted in the literature of the organizational training evaluation. This model provides a straightforward guideline and reduces the measurement demands for training evaluation. The four levels of evaluation criteria are reaction (Level I), learning (Level II), behavior (Level III), and results (Level IV) criteria. This approach emphasizes the progresses from Level I to Level IV, and each level builds on those that come before. The evaluating criteria for each level are outlined as follows.

Level I: reaction is a measure of participants' perceptions to the training. It is basically a measure of client satisfaction.
Level II: learning is a measure of what the participants have learnt and gained from the training.
Level III: transfer is a measure of changes in their behavior when they return to the job after the training program. It also refers to the transfer of training, which measures knowledge and skills gained in the training that are applied on the job.
Level IV: results are a measure of the final outcomes and the change in productivity that occur owing to the contribution of the training.

Although a number of criticism [18–20] and modifications to the model [12, 21, 22] have been suggested and recommended, Kirkpatrick's four-level model of training evaluation continues to be the most prominent and influential among the existing training evaluation frameworks. In the present paper, subjective outcome evaluation findings are reported to evaluate the training program in the P.A.T.H.S. Project. With reference to Kirkpatrick's model as a discussion framework, the first three levels (i.e., criteria of reaction, learning, and behavior) are highlighted. In the model of Kirkpatrick, the reaction evaluation (Level I) and the learning evaluation (Level II) try to measure how the participants react to the training program and what they have learnt and gained, which is also known as subjective outcome evaluation or client satisfaction evaluation. This type of evaluation describes the subjective views of the training participants, which comprise strong parallels with Kirkpatrick's model to measure different training results. Participants' perceived changes in behavior (Level III) were also emphasized in the training programs. Though some comments have criticized that the four-level evaluation model is too simple, this model offers a good starting point as “framework” to formalize the criteria of various outcomes that should be evaluated for a training program.

## 3. Methods

### 3.1. Participants and Procedures

A total of 121 workshops were conducted between 2005 and 2009 (Table 1), with 4,778 participants. Among these 3-day workshops, there were 49 workshops for the Secondary 1, 41 workshops for the Secondary 2, and 31 workshops for the Secondary 3 programs. At the last session of each training workshop, all participants were invited to respond to a structured but anonymous questionnaire (subjective outcome evaluation was the primary evaluation strategy used in this paper). This questionnaire focuses on the perceptions of the participants towards the program content, activities format, program instructors, self-performance, and administrative arrangement. All participants responded to all items in the evaluation form in a self-administration format. Provisions were also made for open-ended responses to enable respondents to make comments of appreciation or provide suggestions on matters not covered by the close-ended questions with predefined answers. Adequate time was given for the participants to complete the questionnaire. After collecting the data, the training team of Project P.A.T.H.S. input the data into an EXCEL file, which is used to compute the frequencies and percentages associated with the different ratings for the items. From 2005 to 2009, a total of 4,167 questionnaires were completed and collected (2,070 for the Secondary 1 level, 1,343 for the Secondary 2 level, and 754 for the Secondary 3 level) (Table 2). The overall response rate was 87.2%.

### 3.2. Instruments

The 31 items of the questionnaire were used to assess the participants' satisfaction with the training program, the instructors, as well as their views towards their own performance. There are several sections in the subjective outcome evaluation questionnaire outlined as follows:

1. participants' basic demographic information,
2. participants' perceptions of the training program, including the program objectives, design, activities format, and interaction among the participants (16 items),
3. participants' perceptions of the instructors, including the understanding of the course, teaching skills, and professional attitude (5 items),
4. participants' perceptions of their own performance, including involvement during program, application of their learning, and having confidence in the project implementation (4 items),
5. participants' perceptions of the administrative arrangement, such as program enrolment, hospitality, venue, and facilities (6 items),
6. things that the participants appreciated most (open-ended question),
7. aspects of the program that require improvement (open-ended question).

## 4. Results

The questionnaire consisted of 31 items with a six-point Likert scale (1 strongly disagree to 6 strongly agree). Reliability analysis shows that the internal consistency of the whole scale was good (alpha 0.96 for the total scale). Besides, all subscales on the training program (16 items), instructors (5 items), participants' own performance (4 items), and administrative arrangement (6 items) were also reliable. The alpha values, mean interitem correlation are presented in Table 3.

The mean number of participants per workshop was 39.5. The total number of evaluation questionnaires completed was 4,167 (Table 2). Among these respondents, 68.7% of them (*n* = 2,863) were female and 31.2% were male (*n* = 1,299), with 5 participants not disclosing their gender. In addition, 72.2% of the participants (*n* = 3,010) were teachers while 26.1% of the participants (*n* = 1,086) were social workers. There were 66 participants from other disciplines such as teaching assistants or program workers. The mean years of the self-reported work experience were 10.5 years, from a minimum of 0 to a maximum of 45 years (*N* = 4,157, SD = 8.18).

Several observations can be highlighted from the quantitative findings based on the closed-ended questions (31 items). First, the participants generally had a very positive perception of the program contents and activities format of the training program (Table 4), including cultivation of participants' positive attitude to adolescent development (97.1%, *N* = 4,040), strengthening of the participants' understanding of positive youth development (97.5%, *N* = 4,056), encouragement of instructors to do their best (97.5%, *N* = 4,053), promotion of the participants' understanding of the Project P.A.T.H.S. including its basic philosophy, design, implementation, and evaluation (97.5%, *N* = 4,061), enhancement of participants' understanding of the Tier 1 Program (97.3%, *N* = 4,043), and strengthening of the participants' understanding of the nature of adolescent development (96.1%, *N* = 4,002). In particular, 97.3% of the respondents (*N* = 4,045) cherished the peer-interaction amongst participants and 93.3% of the respondents (*N* = 3,879) perceived that other participants were satisfied with the training program as well. 

Second, as indicated in Table 5, most of the participants (95.9%, *N* = 3,986) perceived the instructors in a positive and encouraging manner (Table 5). About 98.7% (*N* = 4,102) of the respondents thought that the instructors showed good and professional attitudes. 96.1% (*N* = 4,000) of respondents perceived that the instructors had good mastery of the training curricula and that their teaching was clear and easy to understand (96.4%, *N* = 4,006). Third, regarding the performance of the program participants during the training process, a high proportion of the respondents had positive evaluation of their own performance (97.1%, *N* = 4,035) in the training program (Table 6). For instance, most of the participants perceived that they participated actively during discussion (95.6%, *N* = 3,976). In addition, they reflected that they are willing to apply the specific skills and theories learnt from the training program (97.8%, *N* = 4,065) and having confidence in program implementation after attending the training program (94.4%, *N* = 3,919). Finally, as shown in Table 7, the participants had good evaluation of the administrative arrangement, most of them were satisfied with the administrative arrangement provided (98.7%, *N* = 4,098) and the reception provided by the training team (98.8%, *N* = 4,109). The participants also appreciated the workshop assigned (96.9%, *N* = 4,021) and the venue facilities (96.3%, *N* = 4,005).

## 5. Discussion

The subjective outcome evaluation findings based on the responses of the potential program implementers in the Project P.A.T.H.S. are presented in this paper. According to the evaluation results, the current outcome evidently indicated that the training program had been successfully implemented and the program outcomes were very encouraging. With reference to Kirkpatrick's four-level model, the present quantitative findings showed that the training programs of the project P.A.T.H.S. generally generated positive reactions (Level I), enhanced learning (Level II), and desired behavioral changes (Level III). 

Several observations can be highlighted from the present findings. First, in relation to reaction data (Level I), favorable reactions were found. The training program was well received by the training participants. A large majority of participants reported that the program met their expectations. For instance, the overwhelming majority were satisfied with the training program, its content and format, training instructors, and the overall administration arrangement. As such, it is considered that the first four objectives of the training program have been achieved. In fact, the judgments about the worth and merit of the training program are very critical. If the program implementers were not satisfied with the training experience, they might not buy-in the Project P.A.T.H.S. and would not use what they have learnt in their teaching. They may even advise their colleagues not to buy-in the project and join the training program. Consequently, the well-designed training programs contributed and facilitated the development of positive attitudes among the program implementers towards the P.A.T.H.S. Project. 

With reference to the domain of learning (Level II), the results provide reliable and constructive evidence. From the perspectives of the participants, there is no doubt that the training was tremendously valuable. For example, most of the participants indicated that the program provided sufficient information about the Project, which can help them cultivate their understanding of the nature of youth and positive attitude to adolescent development. In addition, almost all the respondents perceived that the training program strengthened their understanding of positive youth development and Project P.A.T.H.S., including its philosophy, program design, implementation and research. All these positive feedbacks suggest that learning effects were found in the training program. It is believed that the participants could gain knowledge or increase their awareness of the new program implementation. The P.A.T.H.S. training therefore seems to have fulfilled its fifth objectives. In particular, the findings revealed that the knowledgeable and skillful facilitation of the training instructors was highly appreciated and valued. As such, the program implementers could gain an understanding of the humanistic approach to teaching and actual implementation of the Project P.A.T.H.S. though the train-the-trainer workshops. Furthermore, a large majority appreciated the peer-interaction amongst participants. The training workshops actually help them to establish a self-help support network; the interactions among program participants and with the instructor are crucial for social learning. It is believed that the training of Project P.A.T.H.S. provided a supportive environment for the participants to grow and to learn from one another through interaction with other members. This addressed the sixth objective of the training program.

Concerning the behavior and performance change in teacher practice (Level III), the impact should be evaluated after a period of time to ensure the change has occurred. Based on the quantitative findings, it is shown that there were positive influences on the beliefs and attitudes of the participants, and the results suggest that the confidence of the program implementers was boosted. For instance, a high proportion of participants indicated that they are willing to apply the specific skills and theories learnt from the training program. The positive intention of integrating newly learnt knowledge and skills into their practice suggests the support of training transfer and behavior change, and thus students will then benefit from teacher practices. Of course, post-training and implementation research in this context is important.

As indicated in Table 4, many participants agreed that the training program had promoted self-reflection and they expressed that they had confidence in future program implementation. Such reflection could help improve instructional effectiveness and bridge the gap between adopted new philosophy of positive youth development and usual practice. There is a close relationship between self-reflection of teachers and their abilities to integrate theory with practice. It is noteworthy that if program implementers feel confident in implementing a new and unfamiliar program, their resistance will be diminished accordingly, thus empowering program implementation positively. Hargreaves and Fullan [23] argued that teachers' behaviors and beliefs were closely bound together. When teachers were empowered, they would become more involved and satisfied with their job, which in turn has a positive impact on student outcomes. 

This study was aimed to evaluate the training program of the Project P.A.T.H.S. by using subjective outcome evaluation based on the perspective of the training participants. On the whole, the training program can be regarded as successfully implemented, and the training objectives were also well achieved. It also replicated the previous subjective outcome evaluation findings of the training program of the Experimental Implementation Phase [24–28]. Based on the principle of triangulation and utilization-focused evaluation [14], the present findings are consistent with the previous research findings which showed that most of the stakeholders had favorable perceptions of the training program. 

There are several strengths of this study. First, this study investigated different aspects of subjective outcome evaluation, including views of the participants towards the training program, training instructors, perceived effectiveness, and overall satisfaction, and all these scales were found to be reliable. Second, a respectable sample size was used in the study. Actually, it is noteworthy that there are very few scientific studies on the training program of positive youth development programs using such a large sample in the international context. Third, this study is one of the few empirical studies on the pre-implementation training program for the program implementers in a positive youth development program in Chinese context. Therefore, the present study provides a significant contribution to the literature.

However, there are three limitations of this study. First, as only the first three levels of Kirkpatrick's model are discussed in this paper, the discussion is limited. In particular, the use of subjective outcome evaluation findings as support for Kirkpatrick's model should proceed with caution. Second, there is an alternative explanation for the present positive outcomes. Given the demand characteristic (i.e., group pressure or they consciously act in a favorable manner), the participants might give more positive evaluation or tend to focus on the positive side of the training. Nevertheless, this alternative explanation could be partially dismissed since all the participants were professionals and they were invited to reflect their views in a frank way with a serious manner before completing the questionnaire. Moreover, the questionnaires were anonymous in nature. Third, since the present findings are only based on quantitative data, collection of qualitative data with integration of the quantitative findings can give a comprehensive picture about the impact of the training programs. Despite these limitations, the present findings are pioneering addition to the Chinese database on positive youth development. Research studies have consistently showed that the Project P.A.T.H.S. was effective in promoting holistic development in Chinese adolescents [29–37]. With hindsight, it may be conjectured that the positive evaluation findings are a result of the quality training program of the project. 

## Figures and Tables

**Table 1:** Information and attendance statistics of the training program of Project P.A.T.H.S. from 2005 to 2009.

Grade	Secondary 1		Secondary 2		Secondary 3		Total	
Year	No. of participants	No. of workshops	No. of participants	No. of workshops	No. of participants	No. of workshops	No. of participants	No. of workshops
05-06	358	4	NA		NA		358	4
06-07	1,353	28	288	4	NA		1,641	32
07-08	442	10	894	26	197	4	1,533	40
08-09	186	6	311	10	617	23	1,114	39
09-10 (for control schools only)	20	1	19	19	93	4	132	6

Total	**2,359**	**49**	**1,512**	**41**	**907**	**31**	***4,778***	***121***
	***147 days***		***123 days***		***93 days***		***363 days***	

**Table 2:** Information of the respondents.

Grade	Secondary 1			Secondary 2			Secondary 3			Total		
Year	Social workers	Teachers	Others	Social workers	Teachers	Others	Social workers	Teachers	Others	Social workers	Teachers	Others

05-06	108	206	12	NA			NA			108	206	12
06-07	297	867	4	91	142	0	NA			388	1,009	4
07-08	88	306	4	215	612	9	73	102	2	376	1,020	15
08-09	27	128	3	53	198	5	121	380	3	201	706	11
09-10 (for control schools only)	2	14	4	1	13	4	10	42	21	13	69	29

Total	**522**	**1,521**	**27**	**360**	**965**	**18**	**204**	**524**	**26**	**1,086**	**3,010**	**71**
	***2,070***			***1,343***			***754***			***4,167***		

**Table 3:** Mean and standard deviations among the variables by grade.

	S1		S2		S3		Overall	
	M (SD)	*α* (Mean^#^)	M (SD)	*α* (Mean^#^)	M (SD)	*α* (Mean^#^)	M (SD)	*α* (Mean^#^)
Participants' perceptions of the training program (16 items)	4.82 (.64)	.95 (.52)	4.76 (.56)	.94 (.50)	5.11 (.42)	.92 (.41)	4.85 (.59)	.94 (.51)
Participants' perceptions of the instructors (5 items)	5.19 (.76)	.94 (.76)	5.00 (.75)	.94 (.76)	5.59 (.47)	.90 (.65)	5.20 (.75)	.94 (.76)
Participants' perceptions of their own performance (4 items)	4.77 (.57)	.80 (.51)	4.75 (.56)	.83 (.56)	4.90 (.51)	.81 (.53)	4.79 (.56)	.81 (.53)
Participants' perceptions of the administrative arrangement (6 items)	4.94 (.51)	.79 (.40)	4.93 (.52)	.85 (.49)	5.02 (.46)	.84 (.49)	4.95 (.51)	.81 (.44)
Participants' overall satisfaction (31 items)	4.90 (.54)	.96 (.40)	4.84 (.49)	.96 (.41)	5.14 (.37)	.94 (.34)	4.93 (.51)	.96 (.40)

^#^Mean interitem correlations.

**Table 4:** Summary of the views of the participants towards the contents and activities format of the training program.

Participants' views towards the contents and activities format of the training program	Respondents with positive responses (Options 4–6)							
	S1		S2		S3		Overall	
	*n*	%	*n*	%	*n*	%	*N*	%
(1) It has strengthened my understanding of the nature of adolescent development	1962	94.9	1293	96.3	747	99.1	**4002**	**96.1**
(2) It has helped me to cultivate positive attitude to adolescent development	1991	96.4	1301	97.1	748	99.2	**4040**	**97.1**
(3) It has strengthened my understanding of positive youth development, including its concept, design and research	2006	97.1	1300	97.1	750	99.6	**4056**	**97.5**
(4) It has helped me to understand the Project P.A.T.H.S., including its basic philosophy, design, implementation, and evaluation	1996	96.6	1315	98.0	750	99.5	**4061**	**97.5**
(5) It has strengthened me to understand the content of the Tier 1 Program	2002	96.9	1293	96.6	748	99.3	**4043**	**97.3**
(6) It has helped me to acquire the attitude, knowledge and skills that are conducive to the successful implementation of the Tier 1 Program	1936	93.8	1287	96.3	745	98.8	**3968**	**95.5**
(7) It has helped me to establish self-help support network and shared teaching experiences among the program participants	1950	94.5	1247	93.3	716	95.1	**3913**	**94.2**
(8) The training methods and activities are appropriate (e.g. lecture, games, group discussion)	1898	91.9	1251	93.2	746	99.1	**3895**	**93.6**
(9) Training time is appropriate	1715	83.2	1159	86.4	709	94.0	**3583**	**86.2**
(10) It has met my expectation	1817	88.1	1226	91.4	741	98.4	**3784**	**91.0**
(11) Overall speaking, I am satisfied with the training program	1878	91.0	1257	93.7	749	99.3	**3884**	**93.4**
(12) There was much peer interaction amongst participants	2013	97.5	1287	96.0	745	98.8	**4045**	**97.3**
(13) Instructor(s) encouraged participants to do the best	2007	97.5	1297	96.6	749	99.3	**4053**	**97.5**
(14) I think participants are satisfied with the training program	1886	91.4	1246	92.9	747	99.2	**3879**	**93.3**
(15) It has promoted self-reflection	1938	93.8	1268	94.6	749	99.3	**3955**	**95.0**
(16) It has helped me to recognize factors that affect teaching	1935	93.8	1261	94.0	742	98.4	**3938**	**94.7**

*Note. *All items are on a 6-point Likert scale with 1: strongly disagree, 2: disagree, 3: slightly disagree, 4: slightly agree, 5: agree, 6: strongly agree. Only respondents with positive responses (options 4–6) are shown in the table. S1: Secondary 1 level; S2: Secondary 2 level; S3: Secondary 3 level.

**Table 5:** Summary of the views of participants towards program instructors.

Participants' views towards program instructor(s)	Respondents with positive responses (options 4–6)							
	S1		S2		S3		Overall	
	*n*	%	*n*	%	*n*	%	*N*	%
(1) The instructor(s) had good mastery of the curricula	1969	95.3	1279	95.4	752	99.7	**4000**	**96.1**
(2) The instructor(s) understood the needs of participants	1915	92.7	1233	92.1	743	98.8	**3891**	**93.6**
(3) The instructor(s) showed good professional attitude	2036	98.5	1313	98.1	753	100.0	**4102**	**98.7**
(4) The instructor(s)' teaching was clear and easy to understand	1983	96.0	1272	95.1	751	99.6	**4006**	**96.4**
(5) Overall speaking, I have positive evaluation of the instructor(s)' teaching performance	1968	95.3	1267	94.7	751	99.6	**3986**	**95.9**

*Note. *All items are on a 6-point Likert scale with 1: strongly disagree, 2: disagree, 3: slightly disagree, 4: slightly agree, 5: agree, 6: strongly agree. Only respondents with positive responses (Options 4–6) are shown in the table. S1: Secondary 1 level; S2: Secondary 2 level; S3: Secondary 3 level.

**Table 6:** Summary of the views of participants towards themselves.

Participants' views towards themselves	Respondents with positive responses (Options 4–6)							
	S1		S2		S3		Overall	
	*n*	%	*n*	%	*n*	%	*N*	%
(1) I participated actively during discussion.	1982	95.9	1270	94.8	724	96.0	**3976**	**95.6**
(2) I am willing to apply the specific skills and theories learnt from this training program.	2021	97.9	1299	97.0	745	98.8	**4065**	**97.8**
(3) After attending the training program, I had confidence in program implementation.	1909	92.6	1273	95.2	737	97.7	**3919**	**94.4**
(4) Overall speaking, I am satisfied with my performance.	1994	96.6	1298	97.0	743	98.8	**4035**	**97.1**

*Note: *All items are on a 6-point Likert scale with 1: strongly disagree, 2: disagree, 3: slightly disagree, 4: slightly agree, 5: agree, 6: strongly agree. Only respondents with positive responses (Options 4–6) are shown in the table. S1: Secondary 1 level; S2: Secondary 2 level; S3: Secondary 3 level.

**Table 7:** Summary of the views of participants towards administrative arrangement.

Participants' views towards administrative arrangement	Respondents with positive responses (Options 4–6)							
	S1		S2		S3		Overall	
	*n*	%	*n*	%	*n*	%	*N*	%
(1) Information obtained before attending the workshop	1884	91.3	1246	93.3	712	94.4	**3842**	**92.5**
(2) Workshop assigned	1977	96.0	1301	97.3	743	98.5	**4021**	**96.9**
(3) Location of the workshop	1876	90.8	1268	94.8	728	96.6	**3872**	**93.2**
(4) Reception provided by training team (e.g., traffic arrangement, refreshments, etc.)	2053	99.4	1312	98.1	744	98.7	**4109**	**98.8**
(5) Facilities of the venue.	1955	94.6	1302	97.3	748	99.2	**4005**	**96.3**
(6) Overall speaking, I am satisfied with the administration arrangement	2028	98.4	1319	98.6	751	99.6	**4098**	**98.7**

*Note. *All items are on a 6-point Likert scale with 1: strongly disagree, 2: disagree, 3: slightly disagree, 4: slightly agree, 5: agree, 6: strongly agree. Only respondents with positive responses (options 4–6) are shown in the table. S1: Secondary 1 level; S2: Secondary 2 level; S3: Secondary 3 level.
